# Whose property is this? Exploring dynamics between property relations, housing precarity, and working conditions in Pune's red-light district, India

**DOI:** 10.1080/09584935.2025.2494612

**Published:** 2025-05-12

**Authors:** Sutapa Majumdar

**Affiliations:** a LEAD at Krea University, New Delhi, India; b Laws of Social Reproduction Project (ERC project, H2020), Kings College London (Affiliate), London, UK

**Keywords:** Sex work, brothels, property ownership, tenancy rights, rent control, bhade pavti, Pune, Budhwar Peth

## Abstract

Pune's red-light area, the second largest red-light area in Maharashtra after Mumbai and houses close to five thousand cis female sex workers from India, Bangladesh and Nepal. The denial of housing, a basic human right, forces women into unsafe practices, affecting their livelihood and well-being. Using survey data and interviews, the paper examines the precarious living conditions of the sex workers, access to housing and property, tenancy arrangements and political economy of the business. Further the paper discusses how sex workers in Budhwar Peth creatively manage and profit from their living spaces despite facing legal challenges in securing formal property rights. The paper suggests that the housing rights is essential for sex workers for their safety, legal protection, economic stability and overall wellbeing.

## 1. Introduction

In the wake of lndia's economic liberalization in the 1990s in India, rural-urban migration has been accompanied by the erosion of the social housing policies that were prominent in the 1960s and 70s (Gooptu 2011). State and private entities have demolished informal or impoverished communities across the country to unlock the ‘potential’ of urban land values (Weinstein 2013). This has been part of nationwide efforts to ‘cleanse’ cities for urban infrastructure programs, conservation projects, real estate developments and city beautification.

As a result, red-light areas in India have been demolished or reduced in size including Goa's Baina (Shahmanesh et al. 2015), Mumbai's Kamathipura (Seshu and Pai 2022), Nagpur's Ganga Jamuna (Nagpur Today 2020), and Delhi's GB Road (Deccan Chronicle 2016). Despite the United Nations’ Sustainable Development Goals 2030 emphasizing inclusive, safe, resilient, and sustainable settlements with adequate shelter for all, these goals remain distant for many, especially India's sex workers, who are pushed to the impoverished margins of a highly unequal society. India's sex work population is varied in terms of their gender, caste, class, socio-economic positions, levels of education, place and nature of work.

Sex workers are often discussed in contested terms: legalization versus decriminalization (Kotiswaran 2008; Gangoli 1998) trafficking versus sex work (Magar 2012; Kotiswaran 2011; Meyers 2014) and sex work as work versus sex work as exploitation of labour (Kotiswaran 2008, 2011). Much scholarly attention in recent decades has focused on exposing the negative impacts of anti-trafficking efforts on sex workers. While I concur with many of the critiques of the anti-trafficking apparatus, it is time to shift attention to a more specific call for social entitlements for sex workers within a general movement to reform the ITPA. Housing, a basic right is denied to sex workers in India due to their modes of earning. Housing rights are essential for women in sex work as they would feel secure, be able to bargain for better access to other social entitlements, demand better working conditions (such as infrastructural improvements to their brothels), advocate for their health needs, and move more freely in urban spaces with reduced fear of being singled out and harassed for the kind of work they do (Ruparelia, Reddy, and Corbridge 2021). Housing has been an understudied facet of sex worker precarity; few studies in recent years have focused primarily on this aspect of sex workers’ economic situations (see Reed et al. 2011 for an exception). In the given context, this primary research undertaken as part of my post-doctoral work with the Laws of Social Reproduction Project at King’s College London, centers around the important question of ‘how do sex workers in Pune’s red-light district – Budhwar Peth navigate property relations and tenancy structures to shape their economic autonomy, living conditions, work strategies and mitigate legal precarity associated with their work and residency status?’.

The paper argues that the precarious housing and tenancy arrangements in Pune’s red-light district is a dual testimony of economic exploitation and strategic negotiation by the sex workers, revealing a range of contradictions of property relations under India’s rent control system. Despite lacking access to formal property rights, sex workers leverage informal tenancy arrangements, subleasing and kinship-based networks to assert a degree of economic independence and stability. By examining the spatial organization of sex work, tenancy structures, and economic transactions, the study contends that current urban policies and legal frameworks fail to account for sex workers’ lived realities, reinforcing their marginalization instead of ensuring their right to secure housing and economic stability. Using a materialist feminist^1^ lens (Kotiswaran 2006, 2011), I attempt to show the interplay of economic agencies, residential and rental arrangements, ownership of properties and negotiations that sex worker engage in their everyday lives and at times subverting the patriarchal and capitalist structures that governs housing market and property access – with clients, property owners, brothel owners, agents, police and the state. Ultimately, the paper moves beyond moralizing narratives about sex workers and calls for policy reforms toward tenure security and formal recognition of sex workers’ housing and economic rights as a crucial step toward social inclusion and legal empowerment.

The present study is structured around three main empirical questions: First, what is the relationship between residence status and modes of organizing sex work in Pune's Budhwar Peth? Second, what are the range and patterns of ownership and tenancy arrangements, including rent control properties, across different buildings and streets in Budhwar Peth, and their relation to sex work? Third, how do interpersonal relations and informal networks interact to enable impermanent and informal tenancy and residency controls as well as support the business of sex overall in Budhwar Peth?

## 2. Literature review

Over the decades, scholarships have extensively researched sex workers in India. Many studies have focused on the multifaceted structural realities sex workers face, including the role of poverty, caste, and labour migration while delineating women's entry and exit into sex work (e.g., Agrawal 2020; Orchard 2007; Ramberg 2013; Shah 2014; Sahni, Shankar et.al 2008). Other studies have focused on the violence, stigma, marginalization that sex workers combat (e.g., Cornish 2014; Dasgupta 2021; Hussain and Dasgupta 2023; Ghosal et al. 2022) as a consequence of their mode of earning a living. Still others have highlighted the impact the law plays in shaping sex workers’ experiences (e.g., Kotiswaran 2008, 2011, 2023; Lakkimsetti 2020; Ramachandran 2023).

Global sex workers collectives such as Global Network of Sex Work Project focusses on decriminalization of sex work, sex workers rights, safety and oppose laws and policies which discriminate against women and others marginalized groups. In India, sex worker collectives such as Durbar Mahila Samanwaya Committee (DMSC), National Network of Sex Workers (NNSW), All-India Network of Sex Workers (AINSW), and various other community-based organizations and NGOs across the country advocate for a pro-work position and the decriminalization of sex work (Seshu and Pai 2014). These groups focus on community mobilization, empowerment of sex workers, HIV/AIDS prevention, care and support and work in tandem with policy advocates who focus on advancing the sex workers’ access to rights and legal protections (Vijayakumar, Chacko, and Panchanadeswaran 2015; Shah 2012; Kotiswaran 2011; Wijers 2022).

More recently, studies show that most sex workers in India join sex work for economic independence (Walters 2016a, 2016b), to escape from violent marriages (Nataraj and Majumdar 2021), escape from male control (Guha 2024), to provide a better life for their children (Dalla et al. 2020) and to buy and own property for future stability (Kotiswaran 2011). Economic independence not only bolsters women's agency, but it also cements a sense of confidence that in tum enables sex workers to better ameliorate their stigmatized social position (Kabeer 2002; Sahni and Shankar 2022; Swendeman et al. 2015; Walters 2016b).

Theoretically this study draws from Kotiswaran work on ‘sex work essentialism^2^’, an extension of the materialist feminist tradition, well documented in the seminal work of 1981Fortunati (1981), Federici (1974), and Mies (1981). This framework rationalizes the unique identity and agency of women in sex work by asserting the intrinsic qualities of sexual labour that set it apart from other forms of lowly work (Kotiswaran 2011).

While Kotiswaran challenges the traditional view by emphasizing the economic and labor aspects of sex work and critically engage with mainstream legal feminism which often overlooks the material conditions of the women's lives, this study dive into the lived experiences of the sex workers of Budhwar Peth in particular and explores how sex workers navigate property relations and informal tenancy arrangements to assert economic autonomy despite restrictive rent control laws.

Additionally, by incorporating insights from the sex workers collective, the study bridges academic analysis with advocacy efforts that emphasize housing security, legal recognition and social inclusion. In doing so, the study shifts its focus away from moralized debates on sex work toward a more pragmatic examination of housing as a fundamental component of economic agency. The research hopefully will make a significant contribution to legal studies, feminist economics and urban policy since it demonstrates how informal tenancy structures can both empower and constrain sex workers, thus advocating for housing rights which is crucial for integrating sex work and sex workers into the mainstream.

## 3. Methodology and ethics

The present study on property relations among cis female sex workers in Pune, conducted in collaboration with Saheli Sangh, a longstanding organization advocating for sex workers’ rights in Pune. This research was part of the broader ‘Laws of Social Reproduction’ project. Adhering to rigorous ethical standards, the research tools and consent forms were approved by the ethics boards of King's College London, IFMR/LEAD Krea University in Chennai, and Saheli Sangh. Informed consent was obtained from all participants, with their identities anonymized and data protected according to GDPR and the UK Data Protection Act. Broadly, the research consisted of two parts: (1) a door-to-door household survey of the randomly selected buildings where sex work takes place across all the seven lanes in the area and (2) detailed one-on-one in­ depth interviews with various stakeholders, including sex workers who are directly and indirectly involved with sex work and connected with the Budhwar Peth red-light area. Conducted in two phases, the first phase involved mapping the area to capture the diverse sex worker population and identify patterns in residence and work locations. This mapping occurred from late May to early June 2021, followed by the survey of 23 buildings from mid-June to the end of July 2021. Adopting participatory research method, sex workers were involved in sample selection, finalizing the research tools, and conducting the field survey. Supported by Saheli staff, sex workers conducted the survey, leveraging their knowledge of the space and social norms and ensuring easy access to every building.

The quantitative portion of the study used purposive representative sampling to survey 23 buildings, representing 40% of the total 57 buildings in the area, housing approximately 2000 active sex workers. Stratified random sampling was then used to survey 560 female sex workers, capturing structural details of buildings, types of sex work arrangements, and residency statuses. For the qualitative part, in-depth interviews were conducted with 35 stakeholders, including sex workers, shop keepers, local politicians, property lawyers, police officials, an ex-municipal commissioner, long-tern residents of Budhwar Peth, NGO workers and developers. Using structured questionnaire and convenient sampling method, these interviews explored nuanced aspects of sex work dynamics, property ownership, legal barriers, and the impact of gentrification on the area.

## 4. Major Findings

(a) Demographic details

The study surveyed 560 cisgender female sex workers in Budhwar Peth, aged 22–50, whowere actively engaged in sex work and brothel management. These women came from various regions across India and neighbouring countries like Nepal and Bangladesh. The majority were from West Bengal (255), followed by Karnataka (92) and Nepal (82). Local Maharashtrian women were fewer in numbers (29), as they tended to migrate to other states for sex work. Many respondents were either currently married or had previous marital ties, often resorting to sex work after leaving their families and marriages. More than half had never attended schools and around 20% had completed primary education. All respondents came from impoverished rural backgrounds, underscoring their socioeconomic challenges.

Contrary to common perceptions of trafficking, the sex workers of Budhwar Peth mainly arrived through familial and kinship ties or voluntarily for economic reasons, with some coming as *devadasis*^3^ from Andhra Pradesh and Karnataka. Family-based sex work and artistic courtesan identities appear to be less frequent modes of entering the business today than in an earlier era. Most participants entered sex work fully aware of what it entailed, primarily driven by economic necessity. Of the 560 sex workers, 450 had previously worked in the other labour markets before realizing they could earn more in sex work. This finding aligns with a previous study by Sahni and Shankar (2013), which showed that 49.6% of respondents had been part of other informal labour markets before entering sex work. This thus belies the abolitionist claim that all or most sex workers are forcibly trafficked or coerced into sex work.

(b) Spatial organization of sex work in Budhwar Peth

Geographically, Budhwar Peth spans two *peth* (market) areas: Budhwar Peth and Shukrawar Peth. The area consists of eight distinct lanes, each with its unique composition and modes of operation for sex workers. Daane Aali and Tabla Galli are part of Shukrawar Peth, while the other six lanes are within Budhwar Peth.

Daane Aali, situated next to Shukrawar Peth, had the highest number of sex workers, and in terms of size, this lane was the largest in Budhwar Peth. Bengali, Bangladeshi, and Nepali sex workers dominated the entire lane, except for one building, called the ‘Disco Building’, which housed sex workers from Andhra Pradesh and Karnataka. Daane Aali also had a large number of shops and business establishments run by both sex workers and the non-sex worker community. Margi Galli was the second largest and second busiest lane in the area. Both sides of the lane had brothels – comprising of old and new buildings that have been constructed buildings in place of old *wadas.*^4^ Many local non-brothel-based sex workers came to Margi Galli for work.

Nepali and Bengali sex workers occupied Welcome Galli. This lane was the smallest and had a mix of buildings comprising old *wadas* and newly constructed multi – storied buildings. Tabla Galli, known for its old *wadas* was dominated by sex workers from Karnataka. Tabla Galli has historical significance. It was traditionally inhabited by musicians playing for courtesans *(tawaifs* and other artistically trained women who also provided sexual services). Some family-owned musical instrument shops, run by the Kendulkars^5^ caste group, still exist here. The lane also has many non-sex worker families. Dhamdhere Bol was an area for transgender sex workers and mostly consisted of old *wadas.* Bata Galli, a bustling business area with numerous wholesale electric supply shops has a limited number of brothels, mainly occupied by sex workers from Karnataka, Andhra Pradesh, and Maharashtra. Pasodya Vithoba Lane had similar characteristics to Bata Galli and Pasodya Vithoba Temple Galli with limited brothels and mostly sex workers from Karnataka, Andhra, and Maharashtra.

(c) Modes of organising sex work in Budhwar Peth

Three categories of cis female sex workers were found working in the area's brothels: (1) sex workers residing in brothels (with three subtypes), (2) ‘flying’ or commission based, (3) and independent sex workers.

*Brothel-based sex workers:* Brothel-based sex workers worked across three main subtypes of arrangements: ‘*aadhyas’* (meaning those that worked on a 50:50 basis, sharing equal parts of their income with the brothel owner or keeper); ‘cot-based’ sex workers who rented only the space of a cot within the brothel; and short-term or ‘Contract-based’ sex workers. In these arrangements, sex workers resided in specific brothels under the supervision of a *gharwali* (brothel owner or a brothel keeper). Brothel based sex work continues to be popular in Budhwar Peth as it used to be in the past when sex workers mostly found their way through familial networks and kinship ties.^6^

V^7^,a brothel-based sex worker stated:

I came to Budhwar Peth with a couple of my friends from Dhadwad, Karnataka. As I was growing, I saw young women of my village traveling to Mumbai and Pune for work and I always wanted to do the same, coming to these big cities, work, earn, buy my own clothes and makeup and enjoy. I came to Pune and stayed with an aunt from my village, stayed with her like a family and from her I learnt the tricks of the trade. (V, 29 years, Interview date: 29/07/2021)

V reported that sex workers often agreed to work in the brothel on *aadhya* initially to learn about sex work. She stayed with an aunt from her village, a brothel owner, in her initial years which was a common practice for most of the women coming to Budhwar Peth for sex work as these arrangements ensured security and assured income. But there were also women who took an advanced loan from a brothel owner and had no choice but to remain working there until the loan amount had been paid off.

This arrangement was a temporary one for women, said V as once a sex worker became a ‘chapter’ (meaning someone familiar and clever, who understood the practical details of sex work), she tended to shift to independent work, either as a cot-based sex worker or by taking a room on rent by herself or jointly with other sex workers. *Aadhiya* for many, thus, constituted the primary mode of entry and of first organising one's work in a red-light area. *Aadhiya* was also popular as women entered sex work through known network and could work with selected patrons.

S Tai, a Devadasi stated:

‘My time was different as there was no competition among women in sex work. Women acquired more stable regular clients and earned predictable incomes.’ This trend changed after the early days of economic liberalisation in the 1990s when women from Bihar, West Bengal, Bangladesh, Nepal and other northern states started coming to Budhwar Peth for sex work.

For a minority, brothel work was initially arranged in highly exploitative ways. R^8^, for example, a brothel keeper in Budhwar Peth at the time of this study, had first entered sex work in Bhiwandi in Mumbai through the introduction of an acquaintance from her village some twenty years before. R believed that she was neither forced nor sold into sex work but was ‘misinformed’ about her work. In Bhiwandi, she worked for two years as a brothel-based sex worker and the brothel keeper gave her Rs. 15,000 ($180.96) at the time of returning to her village. She believed that she must have earned significantly more than what was given to her in compensation for her work over the preceding two years. When she realized that she would never receive the full earnings she was due under her previous work arrangement, R shifted instead to Budhwar Peth to work independently.

From being *aadhyas,* sex workers usually progressed to be a ‘cot-based’ sex worker in a brothel where they paid a fixed monthly rental amount, between Rs. 3000 ($36) and Rs. 6000 ($72) depending on one's financial capacity and the facilities available in the brothel. The rent was either paid to the *gharwali* or directly to the owner of the brothel if no brothel keeper was employed to watch over them. In a cot-based system, the relationship between the sex worker and the brothel owner or the keeper remained limited to the payment and receipt of rent. Except for the rent amount, all other income remained with the sex worker. Rao, a male brothel keeper in the area and a partner to a sex worker who also a sex workers’ daughter, said that when brothel keepers aged or when they chose to reside outside the area (especially if their children were in no way connected to sex work), they tended to prefer to manage sex workers on cot-basis rent. ‘This is a hassle­ free arrangement where the brothel keeper is guaranteed assured income every month.’

The third arrangement was ‘contractual’, in which sex workers stayed and worked for a contracted period paying a fixed rent to the *gharwali* or the actual owner of the brothel. This arrangement was more common during festival times when the demand for sex work rose. The sex workers or their agents usually identified brothels, paid the contracted amount to the *gharwali* and left the place once their contracted period ended.

*Flying or Commission – based*: These sex workers usually stayed outside the red-light area and came to do sex work for a fixed time in the area. They worked discreetly and avoided being identified and labelled as sex workers. ‘M’ was a flying sex worker and a peer educator for Saheli Sangh^9^ She first worked as a brothel-based sex worker on the *aadhya* system for close to ten years sand eventually moved out in 2011 due to the regular raids in the area.^10^ She returned later to work as a flying sex worker. She came every morning and worked at the Saheli office from 10 am to 2 pm. After work, she did her makeup and solicited customers until 7 pm. She usually took one to two customers per day and left the area by 7.30 pm or 8 pm. She reported that she lived in a residential colony and no one in her area knew that she did sex work. She worked on commission, paying Rs. 30 ($0.36) per client to the brothel keeper and retained for herself whatever other amount she negotiated with the client.

*Independent sex workers:* These women usually rented out a room for themselves in the area and continued working alone or with the help of their lover or husband, who could also be the *dalal* or pimp. Independent sex workers tended to be younger and better able to service multiple clients and to work night shifts. They sometimes went out with clients on contract. These women preferred to take independent rooms on rent. Such rents were comparatively higher than the normal rooms, and independent sex workers often paid Rs. 8000 ($97) per month for basic, small rooms without any fixtures and amenities up to Rs. 15,000 ($181) or more for rooms with amenities like air conditioning, LED lights, music systems and more per month. Such rates when compared with the cot rent and the rent-controlled prices in the area was quite high, and only high earning sex workers could afford them. While a woman paid anywhere between Rs. 3000 ($36.19) and Rs. 6000 ($72.39) per month [that is, Rs. 100 ($1.21) and Rs. 200 ($2.41) per day] for a cot depending on the furnished facilities, the same woman would end up paying nearly double the rent if she decided to take an independent room. M Tai^11^, an older resident of Budhwar Peth, could not afford to take a room on rent in the area as the rent was too high. She said that newer women, especially from West Bengal and Nepal, earned well from sex work; hence, they could pay higher rents and remain independent.

*Emerging modes of organising sex work:* Sex workers in Budhwar Peth were also open to new modes of organising their work. Recent recruits to the job were typically better informed about the realities of urban sex work when they arrived in the area than were earlier generations (Tambe 2009), and fewer of them were escaping violent marriages (see Nataraj and Majumdar 2021). Instead, many of them came for economic independence and the ability to provide well for their family and children. As a result of entering sex work intentionally with specific goals and aspirations, sex workers were increasingly open to modifying their business practices as it suited them – moving out of the red-light areas and settling in city suburbs, using smartphones and internet to sell sex both outside the red-light area and even virtually rather than in person. Some were becoming escorts and permanent, paid sex partners. Such emergent modes of organising sex work were picking up momentum after demonetization and the pandemic, both of which made direct cash transactions and physical interactions more challenging. Thus, at the time of this research, forces other than the pressures of gentrification and urban renewal were also at work transforming the red-light area and the business of sex in Pune.

(d) Residency status and sex workers’ earnings

In Budhwar Peth, the sex workers charge a standard rate for ‘per shot^12^’, which is around Rs. 250 and Rs.300 ($3.00-$3.60). However, sex workers were open to accepting any amount, especially in the post­ demonetization economy (see also Majumdar and Sahni 1981). In the past, an average a sex worker could easily earn anywhere between Rs. 10,000 and Rs. 12,000 ($120.64-$144.77) per month but since the pandemic, that had become difficult to achieve.

Age and work location decided the clientele number. Younger sex workers could charge Rs. 500 ($6) for 10–30 minutes of time, engaging in single or multiple shots during this time span. They charged a minimum of Rs. 4000 ($48) or Rs. 5000 ($60) per night. While older sex workers could earn the same amount for 10–30 minutes of time, they tended to earn less per night-anywhere between Rs. 1000–Rs. 3000 ($12-$36).^13^ In Budhwar Peth, the younger Bengali, Nepali and the North Indian sex workers charged 5000 to Rs. 8000 ($60-$97) per night. Their rate was much higher than the others because these women were open to experimentation and mostly found their customers through agents unlike women of Andhra Pradesh, Karnataka, and Maharashtra who largely solicited independently. The added fee for the work of brokers increased their base rates.

Within a brothel setup, sex workers on a 50:50 arrangement *(aadhya)* divided the entire amount they earned equally with the *gharwali.* If the sex worker was working on a cot basis, she paid only for additional food and alcohol along with a monthly rental fee for the cot. Women working independently had complete autonomy over their rates and any associated charges for the services they offered. Commission-based or flying sex workers charged the lowest rates as they had limited options in acquiring clients. Without a permanent location from which to work, they were mostly on the run while soliciting clients due to the fear of being caught. As a result, they tended to settle for any price and any client that might come their way. This underscores sex workers’ need for secure access to spaces not only to live but also to work and earn their living. Women staying in and working from brothels or working independently had more secure means of getting clients, either through their own contacts or the contacts of the *gharwali* and the agents, and they did not need to depend on only direct solicitation. Residing in the area also helped secure these sex workers a more regular flow of clients. Independent sex workers on the other hand didn't have such support system and were solely dependent on their fixed customers or *malaks^14^* who would cover most of their living expenses and who often held special feelings for the sex workers.

(e) Tenancy arrangements in Budhwar Peth

The rent control laws have exacerbated the problems of inadequate affordable housing and tenant immobility, significantly impacting rental, housing and land markets in cities (e.g., Gandhi, Green, and Patranabis 2022; Tande et al. 2015). In areas like Budhwar Peth, owning high-value property holds little benefit for owners^15^ due to rent control, resulting in minimal income from tenants. Although the Maharashtra Rent Control Act guides tenancy arrangements in Peth, both owners and tenants often flout these rules to maximize profits.

While bypassing the law may offer short-term economic benefits for some sex workers, it has detrimental effects on their living and working conditions. Mutual agreements for redevelopment or partial redevelopment between owners and tenants are challenging, as neither party wants to bear the repair costs. Consequently, tenants continue to live in deteriorating structures with cracks, leakages, water logging, and drainage blockades until the buildings are either declared unfit for living or collapse.

This becomes evident from the following narrative where an old sex worker remains in a dangerous housing condition in fear that she would be asked to vacate if she demanded repairing of the house. This situation reflects the reality of most rent-controlled properties, where owners, often difficult to trace, take little interest in their tenants’ well-being due to the lack of financial incentives to address their demands. An older sex worker, S Tai, stated:

See the roof of my room, I have covered the roof with a plastic sheet to save myself from getting wet during monsoon. I can't keep anything heavy in my room as the floor can't take the weight, but I can’t ask the owner because if l made even one complaint, I would be asked to leave. Where will I go now? I am old and have no support. (S Tai, Age unknown, Interview date: 28/07/2021)

Within the larger context of rent control laws applied to older buildings, Budhwar Peth offers three types of tenancy rental arrangements for the sex workers: (1) *bhade pavti* or rent control; (2) leave and license system; and (3) informal tenancies.

*Bhade pavti* (*rent-controlled*): This is the oldest fonn of tenancy arrangement in the area, and it applies to most of the old *wadas* in the area. In this system, *bhade pavti* means the rent receipt, which is issued in the name of a particular tenant (mostly the sex worker) by the property owner in return for a meagre rent amount, which in most cases is not more than Rs. 100 ($1.20) per month. Section 31 clause (1) of the MRCA of 1947 and revised act of 1999^16^ states that the landlord should furnish a written receipt in return for any amount received from the tenant as decided by concerned parties, they are punishable with a fine. In Budhwar Peth, this rent receipt in most cases does not come directly from the landlord, as it is difficult to trace the actual owner of the property, since a majority of the original owners have now left the area and have given responsibility of their property to managers, agents, or extended family members, who may or may not be directly related to sex work. Hence, where the property owner fails to provide the *pavti,* the tenant pays the rent in court in order to receive a receipt of the payment made and document their payment history.

Informal *bhade pavti* practices benefitted some tenants. The sex workers of Budhwar Peth that have resided for generations on *bhade pavti* system tended to engage in informal transference of tenancy and subleasing practices within their social and familial networks without the knowledge of the legal owners of their buildings. This enabled some women to leverage their social networks to earn rents from the properties they did not technically own, thereby availing a steady source of monthly income, whether directly or through an intermediary. Section 26 of the MCRA clearly states that subleasing and transfer of tenancy without the knowledge of the property owner is not permitted under the act. It also states that it is lawful for a property owner to take money for the grant or renewal of a lease of the premises or to give his/her permission to transfer the lease^17^ to any other person under Section 56(ii). In such cases, the tenant is then legally eligible to take a swn in consideration for waiving or transferring his/her tenancy as per Section 56(i) of the MRCA.^18^

However, such formal practices were rare. These transfer of occupancy operated entirely informally. The long genealogies of lessee to sublessee were a complex phenomenon and it was difficult for a current tenant to ascertain the identity of the legally documented tenant. The legal property owners seldom challenged these informal subleasing practices as many of them were not aware of such negotiations being made in the first place. Since they usually continued to receive their rent payments on time, they were not overly concerned with what the tenant did unless such arrangements created any legal issues for them.

One elderly sex worker, who had been staying in Budhwar Peth for the last forty­ six years, shared that she has long been a *bhade pavti* tenant, and her rent had only increased from Rs. 30 ($0.36) to Rs. 200 ($2.40) in these last 46 years. While on *bhade pavti,* she had been allowing other sex workers to work from her room on a cot basis. At the time of this research, three sex workers stayed on cot basis rent with her, and she received Rs. 4000 ($48) from each of them per month. Thus, she earned Rs.12,000 ($145) from the sex workers in her space while she paid Rs. 200 ($2.40) as rent to the actual property owner, who has never been in sex work.^19^ Such informal subleasing arrangements could thus aid older *bhade pavti* tenants, in particular, in earning some extra income once they were less able to attract and service clients.

In Budhwar Peth, three quarters of the resident sex workers populations staying as *bhade pavti* tenants did not have sufficient documents to prove the status and duration of their tenancy. This group remained the most vulnerable of any among the sex workers in the area. Often, these tenants without rent receipts were forced to leave the area with little to no financial compensation. P Tai stated:

“I am a bhade pavti tenant and have been paying Rs. 40 ($0.48) per month as rent all my life”. Though I have continued to pay the rent, I have never received a receipt for my payment from the owner. I trusted them but now they are asking me to vacate the house, and I refuse to do so. They are offering me some compensation, but I refused as I do not want to leave the area and have no other place to stay. (P Tai, 35 years, Interview date: 21/08/2021)

Thus, while *bhade pavti* produced low rents for some sex workers and additional income for a few others who managed to sublease their spaces, it also generated significant insecurity overall as it incentivized property owners to illegally displace their tenants. According to the Maharashtra Rent Control Act (MRCA), a landlord cannot legally evict a tenant with a *bhade pavti* (rent receipt) who has been paying rent regularly for many years, unless the tenant violates specific provisions such as those outlined in clause (o) of section 108 of the Transfer of Property Act, 1882, or makes permanent structural changes without the landlord's knowledge (section 16, sub-section (c) and clause 1, sub-section (b) of the MRCA). Further in the same section, clause 1 sub section (b) mentions that if a landlord decides to sell the property, they must either compensate each *bhade pavti* tenant or provide an alternate residence in a newly constructed building, contingent on the tenant agreeing to move. However, these entitlements could be accessed if the *bhade pavti* tenants could show tenancy documents. If otherwise, tenants could be forced to leave the area without any compensation. The study revealed that many old *wadas* in Budhwar Peth have been rebuilt or renovated recently, but displaced *bhade pavti* tenants often did not receive compensation for their illegal evictions. Instead, women were threatened and forced to find alternative residences to continue their sex work. Consequently, more than half of the sex worker population lost both their homes and their ability to earn a livelihood as owners sought to circumvent the *bhade pavti* system.

S Tai^20^, an older sex worker, reported knowing a *bhade pavti* tenant who surrendered her tenancy for a rare swn of Rs. 4,00,000 ($4790) from her landlord. Typically, *bhade pavti* tenants do not give up their rooms; instead, they transfer management to another brothel keeper and receive monthly payments as a fixed passive income. Fifteen years ago, S. Tai paid a deposit of Rs. 2,00,000 ($2140) and a monthly rent of Rs. 5000 ($60) to this *bhade pavti* tenant. S Tai too was a brothel keeper in an old *wada,* in the area and shared her experience of running a brothel as a sub-lessee on a *bhade pavti* property for the last fifteen years. The *wada* was initially purchased by an influential brothel owner called RBK^21^ in 1968 who later transferred her claim to the place to one of her relatives as a *bhade pavti*^22^tenant. Fifteen years back, S Tai had paid a deposit of Rs. 2,00,000 ($2140) and a monthly rent ofRs.5000 ($60) to this *bhade pavti* tenant. At the time of the study, her rent had increased to Rs. 12,500 ($150) per month, collected by the tenant's son, who was not involved in the sex business but benefited from the rent-controlled property.

Despite the property being a bhade pavti, S. Tai had to pay a higher rent as lessee and did not receive any rent receipts, making her an illegal occupant under the MRCA. She remained because running the brothel was profitable, even though she had no legal claim to reside or work there. She lacked documentation of her residency or rent payments, preventing her from obtaining formal identification or accessing social entitlements like voter cards, Aadhar cards, PAN cards, bank accounts, or credit lines. S. Tai's case highlights the routine distortion of tenancy laws in Budhwar Peth, resulting in significant consequences for legal recognition, housing security, and access to social benefits for sex workers.

*Informal tenancies and leave and license agreements:* Informal tenancies add another layer to property arrangements when a *bhade pavti* tenant (or her subtenant) further sublet their space. This setup is unique because it provides space for new sex workers who arrive without a place to work. These sex workers can either work independently or under the *bhade pavti* tenant, who might also function as a brothel keeper. This kind of arrangement largely applied to cot-based sex workers within a brothel system, who might rent a cot or a cabin for a specific period of time. The rules of residency were usually set by the immediate owner and might include: no admission of lovers or other family members, no overnight clients, extra charges for consuming gas, electricity, or alcohol etc.

In contrast, leave and license agreements are a more recent phenomenon, offering a formalized rental arrangement. This system is usually explored by young and new Bengali and Nepali sex workers who earn comparatively well. A formal leave and license agreement allowed a sex worker to obtain legally recognized documents, enabling access to various social welfare schemes. These agreements are formalized on legal stamp duty paper and detail the security deposit amount and monthly rent for a fixed period, usually 11 months. While these agreements establish a formal rent contract, not all clauses are strictly followed, with many details settled mutually off-paper through dynamic negotiation.

If a room or flat is rented by a group of tenants, both the security deposit and the rent are higher. For instance, during the research, a single tenant might pay between Rs.6000 and Rs.8000 ($72-$97) per month, while a room for a group of six sex workers might cost as much as Rs.50,000 ($603) or more per month. The legal formalities of leave and license arrangements thus require higher monthly payments compared to options and are feasible only for higher-earning sex workers. Importantly, no agreement could mention the tenant's profession, as renting a space for sex work is illegal in the court of law.

## 5. Conclusion

This article maps out the intricate relationship between housing precarity, informal property relations, economic agency of the sex workers and the modes of organising sex work in Budhwar Peth. The paper begins by contextualizing sex workers’ struggle for housing within the broader context of urban transformation and gentrification and then maps different tenancy structures – revealing how some sex workers both leveraged informal networks to access and profit from living spaces despite not having formal ownership and how the same also left them vulnerable and displaced. This was highlighted in the discussion on how *gharwalis* or the brothel owner and their families could benefit from rent-controlled housing and owned many of the properties in the area, while the other sex workers rarely could own any properties outright.

Several landlords whose *wadas* ran as brothels remained indifferent to upkeep, renovations, and redevelopment, knowing that they stood to profit very little after compensating the tenants in cash or with accommodation in the new building. Sex workers, too, remained silent about the deteriorating state of the buildings they inhabited as they worried, they would not be able to maintain their landowners’ favour, be evicted and thereby lose their livelihood. The poor prospects for accumulating profits often incentivized unscrupulous landlords to displace their long-term tenants using threats or actual violence and providing no compensation or offer of alternative housing. However, this complex web of sublessees simultaneously enabled a few women who found themselves in a position to document their long-term residence in rent-controlled buildings as *bhade pavti* tenants to be able to capitalize on their tenancy status by subleasing the spaces to others for as long as they retained de facto control of the spaces.

The sex workers of Budhwar Peth who participated in this research reported being fearful of rapid change and worried particularly about being excluded from important decision­ making processes. Tenancy rights were often transferred informally in Budhwar Peth to avoid added costs and lengthy bureaucratic processes. Unlike Mumbai and Kolkata, where the rental rates (pagadi^23^ and salami^24^) are transferred to the next occupant, in Pune the transference of *bhade pavti* status was done informally at a lower rate, almost always without the knowledge of the legal owner. A complex web of sub-lessees and intermediaries had thus formed, making it exceedingly difficult for the original owners to capitalize on property that was theirs on paper. Although the law prohibits it, such practices were standard in the area, and most residents acquiesced to such precariously informal arrangements in the name of keeping costs low. On the one hand, in the absence of arrangements for more stable ownership, sex workers rely on a complex web of informal tenurial arrangements whose complexity offers them a certain security and latitude for informal negotiation and tentative socio-economic gains. However, on the other hand, this informal tenure also creates conditions of precarity by foreclosing opportunities to negotiate for more stable and formal rights.

Change in the red-light district unfolded as a form of punctuated equilibrium. For some, the transformations were gradual (as with long-tern *bhade pavti* tenants who saw their monthly rents increase only minutely over many decades). For others, an illegal eviction from their long-term place of residence and work could be sudden and violent, leaving them with no home or means to earn their living. Ironically, both the slowness and the violent suddenness of changes emerged as a consequence of the tangled web of legal and informal ownership and tenancy patterns produced by rent control laws that stymied the prospects of property ownership in older sections of the city. Old buildings housing generations of sex workers proved both unprofitable to upkeep, update, or refurbish, but if the owner could manage (and stomach) illegal evictions of long-tern residents, the land could be rapidly transformed into a renewed cash cow. Such changes were also complicated by the increasing presence of other diverse business establishments in the area beyond brothels that chipped away at the character of the area as a red-light district and made rapid evictions of sex workers potentially more profitable for landowners.

In an overview of the situation, Mr. Mahesh Zagade, the ex-Municipal Commissioner of Pune, opined that ‘lack of will’ and ‘resistance to change’ were some of the key factors that kept Budhwar Peth stuck in its precarious liminality-neither fully formal nor fully informal and always given to the possibility of calamitous illegal evictions. In the shadows of Pune's rent-control system, Budhwar Peth continued attempting to resist the largescale demolitions and rapid new constructions that have clattered up in other parts of urban India. While the particulars of rent-control in Pune could enable temporary and even long-term advantages for certain sex workers, ultimately, without permanent and secure legal rights to the spaces in which they resided and worked, sex workers in the red-light remained at the mercy of unscrupulous and unpredictable property owners seeking ways to wrest back de facto control of buildings they had never themselves occupied. Greater attention to dissolving a reliance on informal tenancy agreements while simultaneously securing sex workers permanent property rights is a necessary next step in the fight to achieving sex workers’ full citizenship and dignity in India. Rather than focus more abstractly on ‘rights of sex workers’ (see Lakkimsetti 2020) or on a moralizing approach to rescue (see Magar 2012), the implication of the present study is that policy should rather recognize the economic motives of sex workers and intervene in issues of tenure and ownership that actually address the goals and desires of the most precariously perched sex workers. With secure access to a place to live and improved workspaces in which to safely earn money, sex workers in India will be much better positioned to negotiate for increased power, social recognition, and dignity in other aspects of their lives. Stable housing is not just a necessity but a fundamental right that is central to sex workers’ dignity, financial stability and full citizenship.

## Notes

1 Rooted in Marxist and socialist feminism, materialist feminist approach examines women’s oppression through the lens of economic relations, social structure, labour exploitation and property ownerships. This theoretical framework states that capitalism and patriarchy together exploits women’s labour and calls for systemic change in labour relations and economic structures to dismantle gender oppression at its root.

2 Sex work essentialism critiques the tendency to view sex work as having an inherent, unchanging nature (exploitation, victimhood, moral degradation) that supposedly define all instances of sex work regardless of context. Kotiswaran states that by treating all sex work as essentially the same, one oversimplifies the diversity and obscure the contextual realities of sex workers’ lives which is limiting.

3 Sex worker identities rooted in caste and religious dedication (see Orchard 2007; Ramberg 2013).

4 *Wadas* are squared residential structures, mostly made of wood and stones with a central podium. Usually, members of one cast or members of one family lived in one *wada.* Each Wada was divided on the basis of caste lines in the past along with strict rules of mobility and abided by strict ruled of purity and pollution

5 Kendulkar used to play musical instruments for the performers residing in Bavankhani (a building with fifty-two windows), who entertained the erstwhile nobility. By caste they are Saiva Brahmin, worshipper of Lord Shiva.

6 Tambe (2006), describing brothel life in 1920's Bombay, demonstrates that brothels functioned as 'alternate residences', especially for those sent away by their families. She notes that actual and fictive kinship ties worked to legitimise the participation of young women in sex work by conforming to the rules of hierarchy, loyalty and obedience of the subordinates.

7 Interview with V on 29th July 2021 at Saheli office, Budhwar Peth, Pune.

8 interview with R Tai (sister), a brothel keeper in Margi Galli on 5th August 2021, Budhwar Peth, Pune.

9 Interview conducted on 8th August 2021 at Saheli Sangh office in Budhwar Peth, Pune.

10 http://archive.indianexpress.com/news/sex-workers-allege-excesses-in-police-raid-to-submit-evidence-to­commissioner/739326/

11 Interview was taken at Saheli Office, Budhwar Peth, on 9th August 2021.

12 Per shot is defined as one act of penetrative sex with the client resulting in ejaculation.

13 These rates were mentioned by different respondents of the research during the study.

14 Malaks form affective partnerships with sex workers. They usually begin as regular customers and eventually they develop special feelings. For the women too, *malaks* become more than a customer and close to a lover or a husband. TI1ese men could be depended on to provide major financial contributions to the household. *Malaks* may also become fathers of sex worker children or may identify themselves as such when required. Malaks may also be married with a wife and children outside the red-light area, shouldering some financial responsibility for both households.

15 The Maharashtra Rent Control Act (MRCA) 1999 provides protection to tenants who have been staying for three to four generations, and the owners can only evict them in return for heavy compensation, which is difficult to arrange.

16
https://lj.maharashtra.gov.in/Site/Upload/Acts/l-Maharashtra%20Rent%20Control%20Act-l999.pdf

17 In cities like Mumbai, for the transfer of tenancy, around 33% of the sum in the transaction is paid in cash to the landlord to influence the transfer of tenancy or assignment. Unlike Mumbai, those on bhade pavti or on rent contr·ol properties in Pune's red light area do not transfer their tenancy rights but informally sublease their place of stay to a known lessee (without the knowledge of the landlord) as informed earlier in return of a lumpsum amount at the beginning and monthly rent thereafter and use the property to live or run it as a brothel.

18
https://blog.ipleaders.in/maharashtra-rent-contro1-act-analysis-loopholes-ways-bridge-gap/

19 Bhade Pavti, tenant of an old wada in Pasodya Vithoba Temple, Bndhwar Peth. The Interview was taken on 22.07.2021.

20 Interview was taken on 28th July 2021 at the Saheli office in Budhwar Peth.

21 Details could be found at https://pune.gov.in/service/view-property-card

22 S Tai could not tell me the exact bhade *pavti* rent paid to the property owner.

23 Pagadi is a traditional tenancy rental model in India where the renter is also part owner of the house (not the land). He enjoys nominal rates as compared to the market rates for rent and has both subletting and selling rights of the property.

24 Like the Pagadi system, the actual owner of the property gives away his room, house to a tenant in return of the lumpsum. This model is quite common in West Bengal, where tenants would occupy a property for a long term and in the process can sublet the property considering himself as the owner and can ask for a share of the selling amount from the owner to vacate the place.
